# Engineering oleaginous yeast *Rhodotorula toruloides* for overproduction of fatty acid ethyl esters

**DOI:** 10.1186/s13068-021-01965-3

**Published:** 2021-05-08

**Authors:** Yang Zhang, Jie Peng, Huimin Zhao, Shuobo Shi

**Affiliations:** 1 Beijing Advanced Innovation Center for Soft Matter Science and Engineering, College of Life Science and Technology, Beijing University of Chemical Technology, Beijing, 100029 China; 2 Department of Chemical and Biomolecular Engineering, University of Illinois At Urbana-Champaign, Urbana, IL 61801 USA

**Keywords:** Fatty acid ethyl esters, Wax ester synthase, *R. toruloides*, Enzyme engineering, Biodiesel, Metabolic engineering

## Abstract

**Background:**

Production of biofuels and green chemicals by microbes is currently of great interest due to the increasingly limited reserves of fossil fuels. Biodiesel, especially fatty acid ethyl esters (FAEEs), is considered as an attractive alternative because of its similarity with petrodiesel and compatibility with existing infrastructures. Cost-efficient bio-production of FAEEs requires a highly lipogenic production host that is suitable for large-scale fermentation. As a non-model oleaginous yeast that can be cultured to an extremely high cell density and accumulate over 70% cell mass as lipids, *Rhodotorula toruloides* represents an attractive host for FAEEs production.

**Results:**

We first constructed the FAEE biosynthetic pathways in *R. toruloides* by introducing various wax ester synthase genes from different sources, and the bifunctional wax ester synthase/acyl-CoA-diacyglycerol acyltransferase (WS/DGAT) gene from *Acinetobacter baylyi* was successfully expressed, leading to a production of 826 mg/L FAEEs through shake-flask cultivation. We then mutated this bifunctional enzyme to abolish the DGAT activity, and further improved the titer to 1.02 g/L. Finally, to elevate the performance of Δ*ku70*-AbWS* in a bioreactor, both batch and fed-batch cultivation strategies were performed. The FAEEs titer, productivity and yield were 4.03 g/L, 69.5 mg/L/h and 57.9 mg/g (mg FAEEs/g glucose) under batch cultivation, and 9.97 g/L, 90.6 mg/L/h, and 86.1 mg/g under fed-batch cultivation. It is worth mentioning that most of the produced FAEEs were secreted out of the cell, which should greatly reduce the cost of downstream processing.

**Conclusion:**

We achieved the highest FAEEs production in yeast with a final titer of 9.97 g/L and demonstrated that the engineered *R. toruloides* has the potential to serve as a platform strain for efficient production of fatty acid-derived molecules.

**Supplementary Information:**

The online version contains supplementary material available at 10.1186/s13068-021-01965-3.

## Background

The global energy demand and environmental concerns have attracted worldwide attention to green and sustainable energy sources [1]. As one of the most promising alternative energy sources, biodiesel has high energy density, and is compatible with current infrastructure [2]. Moreover, it showed several advantages over petrodiesel, such as higher lubricity, and lower tailpipe emissions [3, 4]. Biodiesel consists of long-chain alkyl esters, mainly fatty acid methyl esters (FAMEs) and fatty acid ethyl esters (FAEEs) [5]. Currently, commercial strategies for biodiesel production mainly use plant oils or animal fats as feedstock to produce FAMEs through chemical transesterification [6, 7]. However, these strategies compete for lands and materials required for food production and also cause environmental problems [2, 8]. Therefore, it is desirable to use renewable plant biomass as a feedstock to produce FAEEs by microbial fermentation [9].

Over the past decades, various microbial hosts such as *Escherichia coli*, *Saccharomyces cerevisiae*, and *Yarrowia lipolytica* have been engineered to produce FAEEs. In *E. coli*, the production of FAEEs was achieved by heterologously expressing genes encoding the pyruvate decarboxylase (PDC), alcohol dehydrogenase (ADH), and wax ester synthase/acyl-CoA-diacyglycerol acyltransferase (WS/DGAT) gene from *A. baylyi* [5]. The maximum FAEEs titer reached 19 g/L by adding exogenous oleic acids in fed-batch pilot-scale fermentation [10]. Röttig and coworkers showed that use of a mutant AbWS from *A. baylyi* (Ile355Gly) led to a higher FAEEs titer than the wild-type AbWS in *E. coli* [11]. In *S. cerevisiae*, Shi and coworkers constructed a similar FAEE biosynthetic pathway and demonstrated the WS from *Marinobacter hydrocarbonsticus* led to the highest FAEEs titer of 6.3 mg/L [12]. Later, Yu and coworkers improved the titer to 0.52 g/L FAEEs by adding exogenous fatty acids [13]. In both *E. coli* and *S. cerevisiae*, the production of FAEEs was regarded to be limited by the supply of lipid precursors, and the addition of exogenous oleic acids can improve the final titer significantly [10, 13], which suggests that use of host strains with robust lipid producing capacity such as oleaginous yeasts may be advantageous. Indeed, Xu and coworkers engineered a representative oleaginous yeast *Y. lipolytica* to produce FAEEs and found that the subcellular localization of WSs was essential for the production of FAEEs in the strain [14]. Cytosolic expression of WS from *A. baylyi* ADP1 only resulted in marginal production at 7.1 mg/L, and when WS was targeted to the endoplasmic reticulum (ER), the FAEEs titer was improved significantly to 136.5 mg/L. In a further study, Gao and coworkers revealed that the expression of WS from *M. hydrocarbonsticus* led to the highest FAEEs titer of 0.4 g/L with additional exogenous ethanol, and through pathway optimization for supplying cytosolic acyl-CoA, the production titer reached 1.18 g/L [15].

In addition to *Y. lipolytica*, *R. toruloides* is another promising oleaginous yeast. Compared with *Y. lipolytica*, *R. toruloides* produces a higher titer of lipids [16, 17], and can naturally utilize a variety of carbon sources derived from plants, including xylose and cellobiose [18], thereby exhibiting a broader industrial application prospect in the production of lipid-related products. However, metabolic engineering of *R. toruloides* was hindered by the lack of efficient genetic manipulation tools [16] [19], and only limited metabolic engineering efforts were reported [16, 18]. To date, there is no report using this species to produce biodiesels using a metabolic engineering approach.

**Fig. 1:**
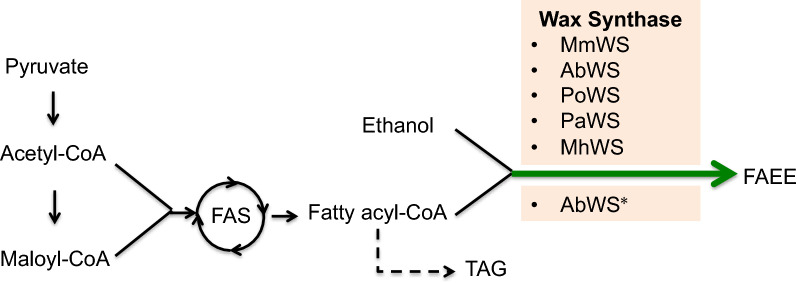
Construction of the FAEE biosynthetic pathway by introducing WSs sourced from various organisms

In the present study, we explored the potential of *R. toruloides* to produce FAEEs. Five WSs previously reported in *S. cerevisiae* were codon optimized and introduced to *R. toruloides* (Fig. 1) [12]. However, only the AbWS from *A. baylyi* was successfully expressed, and the recombinant strain successfully produced FAEEs by adding exogenous ethanol to the culture. Then, the AbWS was engineered to improve the production of FAEEs by abolishing its side activity. Finally, by carrying out fed-batch fermentation in a 1-L fermenter, the production reached a maximum of 9.97 g/L FAEEs (including both extracellular and intracellular titers). To the best of our knowledge, this engineered cell factory possessed the highest FAEEs production levels in a yeast host. Notably, the FAEEs produced by *R. toruloides* were mainly secreted outside the cell, which would greatly reduce the cost in the subsequent downstream processes.

## Results

### Overexpression of WSs for FAEEs production in *R. toruloides*

WS catalyzes esterification of fatty acyl coenzyme A (fatty acyl-CoA) with alcohols of various chain lengths for synthesizing fatty esters. WSs derived from different sources have a wide range of substrate preferences, leading to different catalytic efficiencies. When it is expressed in a heterologous host, the source of the enzyme is a key factor to affect the catalytic efficiency.

To compare the efficiency of various WSs in *R. toruloides*, five previously reported wax ester synthase genes [12] were selected and codon optimized. They were MhWS from *M. hydrocarbonoclasticus* DSM 8798 (GenBank ID: EF219377.1), AbWS from *A. baylyi* ADP1 (GenBank ID: AF529086), RoWS from *Rhodococcus opacus* PD630 (GenBank ID:GQ923886), MmWS from *Mus musculus* C57BL/6 (GenBank ID: AY611032), and PaWS from *Psychrobacter arcticus* 273–4 (GenBank ID: YP_263530). Additional file 1: Table S1 summarizes the application of these enzymes in *S. cerevisiae* and their specific enzyme activities for FAEE production.

**Fig. 2:**
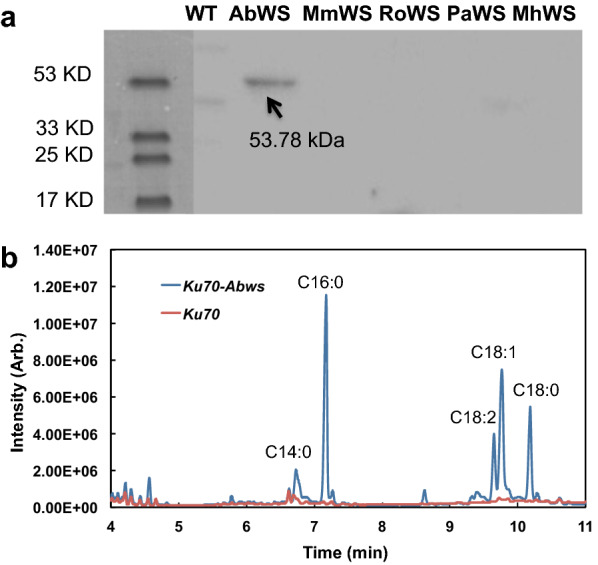
Overexpression of wax synthase genes from different sources in *R. toruloides*. **a** Detection of protein expression by Western blot. “WT”, “AbWS”, “MmWS”, “RoWS”, “PaWS” and “MhWS” represent the protein samples extracted from *R. toruloides* Δ*ku70*, Δ*ku70*- *AbWS*, Δ*ku70*- *MmWS*, Δ*ku70*- *RoWS,* Δ*ku70*- *PaWS* and Δ*ku70*- *MhWS*, respectively. **b** GC–MS analysis of the FAEEs produced in the recombinant Δ*ku70*-*AbWS* strain (the blue line) and the parental strain *R. toruloides* Δ*ku70* (the red line). The components of C14:0, C16:0, C18:0, C18:1 and C18:2 stand for ethyl myristic, ethyl palmitic, ethyl stearic, ethyl oleic, and ethyl linoleic, respectively

To construct the recombinant plasmids, a Flag tag was added at the start of each gene and subcloned into plasmid pKOCAR2. The resulting plasmids were transformed to *R. toruloides* Δ*ku70*, yielding the recombinant strains named Δ*ku70*-*MhWS*, Δ*ku70*-*AbWS*, Δ*ku70*-*RoWS*, Δ*ku70*-*MmWS* and Δ*ku70*-*PaWS* (Table 1). The expression of WSs was examined by Western blot method. It was found that only AbWS with a molecular weight of 53.78 kDa was successfully expressed (Fig. 2a), with a band around 53 kDa obtained. Therefore, the recombinant Δ*ku70*-*AbWS* was used as the candidate for further research.

**Table 1:** Strains and plasmids used in this study

Strains or plasmids	Relevant characteristics	Source or reference
Strains		
Δ*ku70*	*R. toruloides* IFO0880 deficient in *ku70*	[34]
Δ*ku70-AbWS*	Δ*ku70* strain harboring the *AbWS* cassette	This study
Δku70-AbWS*	Δ*ku70* strain harboring the AbWS* cassette	This study
Δ*ku70-MmWS*	Δ*ku70* strain harboring the *MmWS* cassette	This study
Δ*ku70-MhWS*	Δ*ku70* strain harboring the *MhWS* cassette	This study
Δ*ku70-PaWS*	Δ*ku70* strain harboring the *PaWS* cassette	This study
Δ*ku70-RoWS*	Δ*ku70* strain harboring the *RoWS* cassette	This study
AGL-1	*A. tumefaciens* strain AGL0 recA::bla pTiBo542ΔT Mop^+^ CbR	[15]
DH5α	*E. coli* (*sup*E44 *lac*U169 *hsdR*17 *rec*A1 *end*A1 *gyr*A96 *thi*-l *rel*A1)	Laboratory storage
Plasmids		
pKOCAR2	Harboring the hygromycin selection cassette for target gene incorporation at the specific site in *CAR2* gene	[32]
p101	Harboring the *MhWS* cassette	This study
p102	Harboring the *AbWS* cassette	This study
p103	Harboring the *RoWS* cassette	This study
p104	Harboring the *MmWS* cassette	This study
p105	Harboring the *PaWS* cassette	This study
p1022	Harboring the AbWS* cassette	This study

The strain was cultured in 10 mL fermentation medium in an orbital shaker at a rotary rate of 250 rpm at 30 °C. Since *R. toruloides* is unable to produce ethanol under laboratory conditions, exogenous ethanol was added to the culture medium. To minimize the inhibitory effect of ethanol, the strain was cultured by 8 ~ 12 h (OD600 reached to ~ 2) before feeding ethanol. To facilitate the extraction process, 10% dodecane was added to the culture as reported previously [15], and ethyl heptadecanoic was used as the internal standard. Extracellular FAEEs were extracted to the dodecane layer and detected by gas chromatography–mass spectrometry (GC–MS) (Fig. 2b). The composition of FAEEs produced by Δ*ku70*-*AbWS* was mainly composed of ethyl myristic (C14:0), ethyl palmitic (C16:0), ethyl stearic (C18:0), ethyl oleic (C18:1), and ethyl linoleic (C18:2). The ratio of each component resembled that of total lipid in the same strain (Additional file 1: Fig. S1).

### Exploration of the optimal exogenous ethanol concentration for FAEEs production

**Fig. 3:**
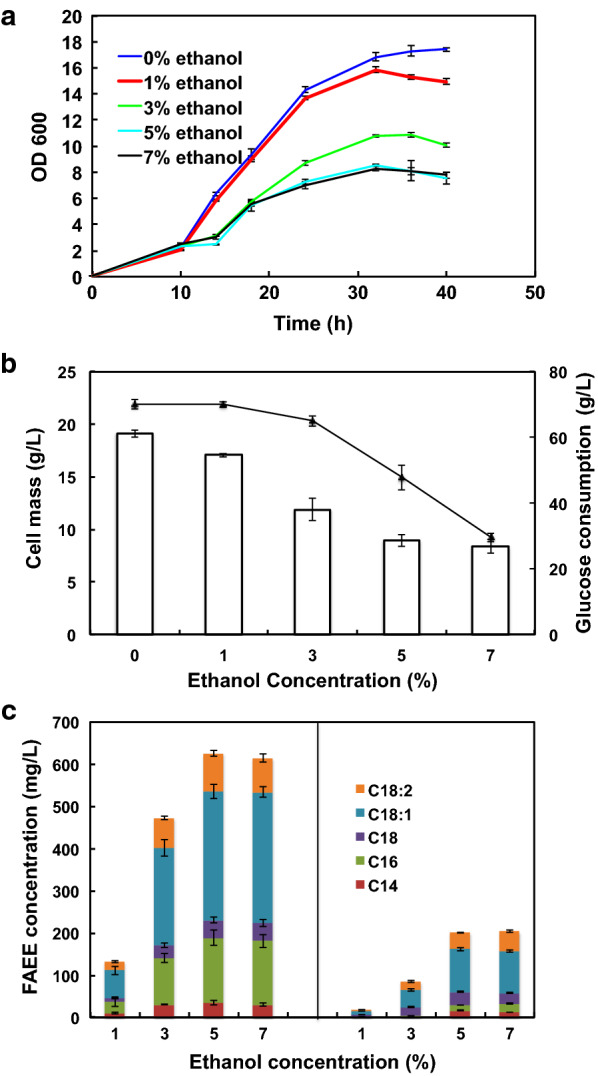
Effect of ethanol on growth and FAEE production of Δ*ku70*-*AbWS*. **a** Growth curves of Δ*ku70*-*AbWS* under different ethanol concentrations. **b** Effects of different ethanol concentrations on the final cell mass formation and the glucose consumption. The bars represent cell mass and the lines represent the amount of consumed glucose. **c** The effect of ethanol on FAEE production titers. The left panel shows extracellular FAEEs titers and the right panel shows intracellular FAEEs titers. C14:0, C16:0, C18:0, C18:1 and C18:2 stand for ethyl myristic, ethyl palmitic, ethyl stearic, ethyl oleic and ethyl linoleic, respectively

Although ethanol is required for FAEEs production, its toxicity may affect cell growth and lead to a decreased FAEEs yield. Therefore, we sought to determine an optimal ethanol concentration for both minimizing the substrate cost and maximizing the production titer. Ethanol was added to the medium at a final concentration of 8.0 g/L (1%), 24.0 g/L (3%), 40.0 g/L (5%), and 56.0 g/L (7%), respectively. Figure 3a shows the effect of different ethanol concentrations on cell growth of the recombinant Δ*ku70*-*AbWS* strain in shake-flask fermentation. As expected, ethanol had an inhibitory effect on cell growth. When the ethanol concentration was 1%, the effect on cell growth was inconspicuous. But when the ethanol concentration was above 3%, the effect on cell growth was significant. The addition of ethanol also had a similar effect on the final cell mass. Figure 3b shows the effect of different ethanol concentrations on cell mass formation and glucose consumption. When the ethanol concentration was above 3%, the final cell mass almost decreased by half, and the glucose consumption was significantly reduced.

We then measured the FAEE titer in shake flask cultivation. After culturing for 60 h, the extracellular and intracellular FAEEs were extracted by different methods, and the results are shown in Fig. 3c. When the ethanol concentration was 1% and 3%, the extracellular FAEEs titers were significantly lower than that of 5% and 7%. A similar trend was also noted for the production of intracellular lipids analyzed by thin layer chromatography (TLC) (Additional file 1: Fig. S2). When the ethanol concentration was 1% and 3%, the fatty acid precursor was mainly stored in the triglycerides (TAG) form, and when the ethanol concentration rose to 5% and 7%, most of the fatty acid precursor flowed to the synthesis of FAEEs. Taken together, the maximum FAEEs titer was 826 mg/L, including 625 mg/L extracellular and 201 mg/L intracellular titers, under the 5% exogenous ethanol condition. When the ethanol concentration was further increased to 7%, the FAEEs titer did not show a further increase. Thus, 5% is considered as the optimal ethanol concentration for FAEEs production. It is worth mentioning that the background strain Δ*ku70* also appeared to produce a small amount of extracellular FAEEs after adding exogenous ethanol (Additional file 1: Fig. S3). This is possibly due to the existence of native lipases in the strain, which can catalyze the esterification process [20].

To further improve the FAEE titers, we also tried a nitrogen-limited medium (70 g/L glucose, 0.75 g/L yeast extract, 1.7 g/L yeast nitrogen base without amino acids and ammonium sulfate, and 0.1 g/L (NH_4_)_2_SO_4_, pH 5.6) for shake-flask cultivation. The medium was reported to improve lipid content in *R. toruloides* [21]. However, the FAEE titer was lower than that in the fermentation medium (Additional file 1: Fig. S4). Thus, we used the fermentation medium for subsequent studies.

### Modification of the AbWS enzyme to improve the FAEEs production

**Fig. 4:**
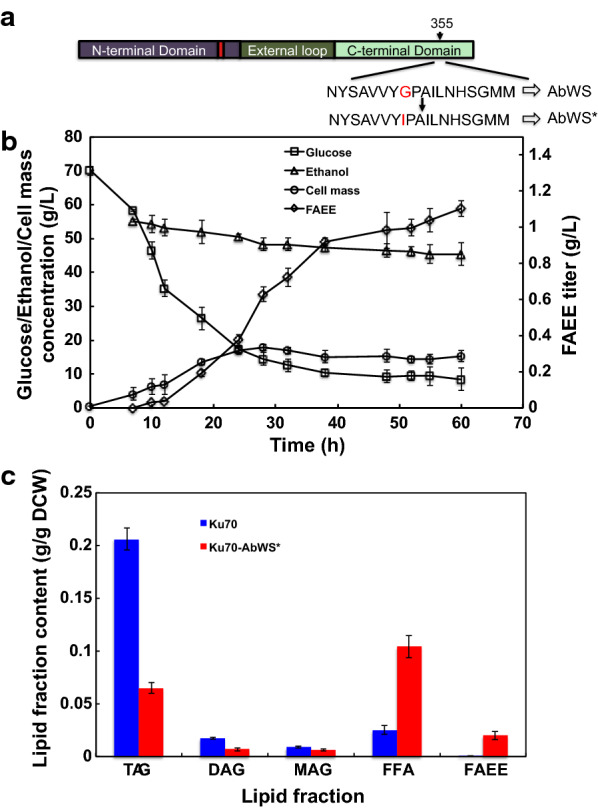
Modification of the AbWS for improving the FAEEs production. **a** Point mutation of AbWS by substituting the glycine to isoleucine at position 355. **b** Time-course profiles for Δ*ku70*-AbWS* by shake-flask cultivation with 5% ethanol. **c** Comparison of intracellular lipid contents in Δ*ku70* and Δ*ku70*-AbWS*

As reported previously, a point mutation of AbWS at residue 355 from glycine to isoleucine led to a shifted substrate selectivity toward shorter chain alcohols and an impaired DGAT activity [11]. Guided by this finding, we created the same mutant AbWS* (Fig. 4a) and introduced it to the Δ*ku70* strain. To compare the efficiency with the wild-type AbWS, the AbWS* and AbWS expression cassettes were integrated in the same CAR2 site of the chromosome to reduce discrepancies caused by integration sites. Only white colonies were selected that indicated as a correct integration (Additional file 1: Fig. S5). Gene sequencing was used to further verify the integration site of the cassettes using the primers of UCar2F/DCar2R (Additional file 1: Table S2) that located in the upstream of Car2L and downstream of Car2R in the chromosome of *R. toruloides*, respectively.

To evaluate the performance of AbWS* and AbWS, the specific activity of the cell lysate was measured. It was found the highest activity of the AbWS* towards palmitoyl coenzyme A was 2.40 ± 0.15 nmol/mg/min, and the highest activity of AbWS was 2.14 ± 0.23 nmol/mg/min. This result was consistent with previous studies [11, 22, 23], which indicated that the point mutation did not improve the specific activity significantly, but rather altered its affinity to ethanol.

The performance of the Δ*ku70*-AbWS* was then tested by shake-flask fermentation. After culturing for 60 h, the extracellular and intracellular FAEEs were measured, respectively, and the production titers are shown in Additional file 1: Fig. S6. Compared with the wild-type AbWS, the mutant showed higher efficiency in producing FAEEs. The maximum FAEEs titer reached 1024 mg/L under the addition of 5% exogenous ethanol, including 810 mg/L extracellular and 214 mg/L intracellular titers. In addition, when ethanol concentration varied from 1 to 7%, compared to Δ*ku70*-*AbWS*, the recombinant Δ*ku70*-AbWS* strain tended to synthesize more ethyl palmitic and less ethyl linoleic. Additional file 1: Fig. S7 displays the proportion of FAEE components under 5% ethanol concentration in both Δ*ku70*-*AbWS* and Δ*ku70*-AbWS* strains. This result might indicate a preference of the AbWS* towards palmitoyl-CoA, although it has not been reported in previous studies.

To further investigate the production yield and productivity, the growth of the strain was monitored along the time course under 5% ethanol conditions. The concentrations of glucose, ethanol, cell mass and FAEE were determined by fermentation. As shown in Fig. 4b, the glucose was consumed by approximately 60 g/L, and the ethanol was decreased from 56 g/L to ~ 45 g/L in 60 h, with a maximum FAEEs titer of 1.10 g/L. The FAEE productivity and yield of FAEEs were 18.3 mg/L/h and 17.9 mg/g glucose. We also monitored the ethanol reduction in a blank medium in an orbital shaker at a rotary rate of 250 rpm at 30 °C, and found that the ethanol concentration was decreased by ~ 10 g/L in 60 h. In comparison, the ethanol concentration was decreased by ~ 11 g/L reduction of during the fermentation process (Fig. 4b). Thus, we considered the consumption of ethanol was mainly due to evaporation.

Both FAEE and TAG were biosynthesized from the fatty acyl-CoA precursor. The intracellular lipid contents of Δ*ku70* and Δku70-AbWS* were measured after shake flask cultivation. As shown in Fig. 4c, in the parent Δ*ku70* strain, the lipids were stored mainly in TAG form (~ 0.2 g/g DCW), while in Δku70-AbWS*, the TAG contents decreased significantly (~ 0.06 g/g DCW), and the intracellular FAEEs increased greatly. To our surprise, the FFAs in the recombinant strain was also increased, indicating the expression of AbWS* may induce the decomposition of TAG.

### Overproduction of FAEEs during high-density cultures using bioreactors

**Fig. 5:**
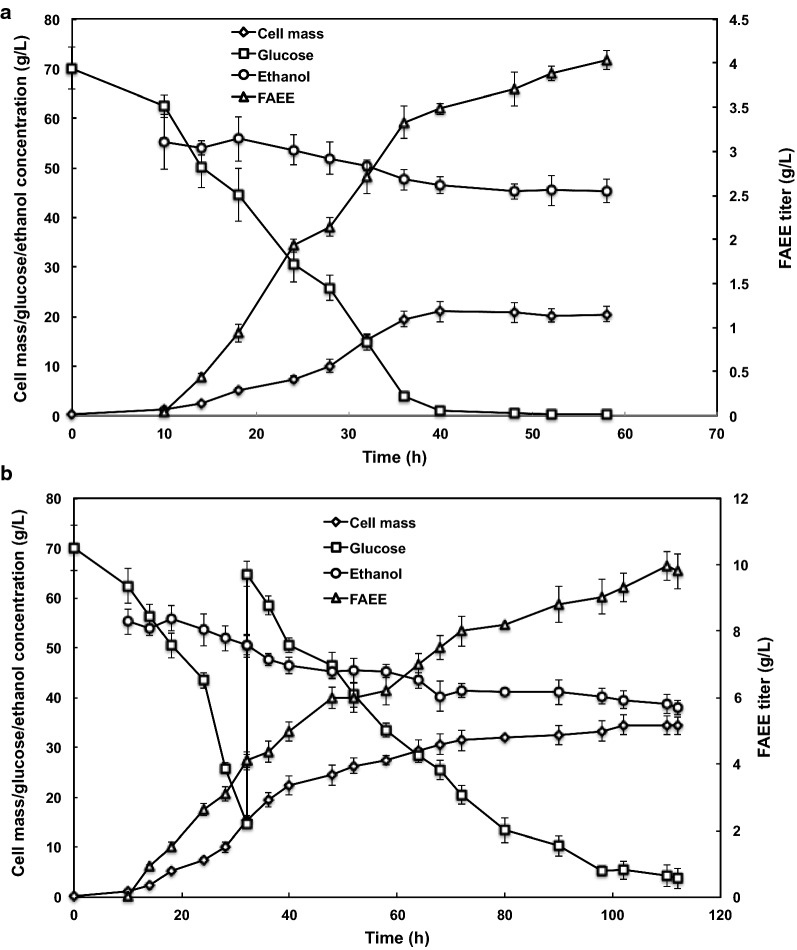
Time-course profiles for batch (**a**) and fed batch (**b**) cultivation of *R. toruloides*. Empty square, glucose concentration; black diamond, cell mass; empty triangle, FAEE concentration; black circle, ethanol

To evaluate its performance in a bioreactor, we cultivated the Δ*ku70*-AbWS* strain in a 1-L bioreactor under aerobic batch (Fig. 5a) and fed-batch (Fig. 5b) conditions, respectively. The concentrations of glucose, ethanol, FAEEs and cell mass were all monitored over time.

We first tested the performance of Δ*ku70*-AbWS* through batch cultivation. The initial glucose concentration was 70 g/L, and the medium was inoculated with 10% seed culture. As shown in Fig. 5a, when the fermentation was finished at 58 h, there was a total consumption of ~ 10 g/L ethanol, and the final titer, productivity and yield of FAEEs were 4.03 ± 0.11 g/L (including an intracellular titer of 0.52 g/L), 69.5 mg/L/h, and 57.9 mg/g (g FAEEs produced/g glucose consumed), respectively. Compared with shake-flask batch fermentation, the FAEEs titer was significantly improved in the bioreactor, and this may be benefited from the continuous ventilation and the appropriate pH in the bioreactor. In addition, the pattern of feeding ethanol may also contribute to the higher titer of FAEEs because the ethanol was pumped to the bioreactor, which may decrease the toxic effect to the cells.

To further improve the FAEE production, a fed-batch strategy was employed. As shown in Fig. 5b, after culturing for 32 h, the glucose concentration was decreased to 14 g/L. To avoid carbon limitation, additional glucose was fed to the 500 mL medium in the bioreactor, leading to a concentration of 64 g/L. When the fermentation was finished at 110 h, there was a total consumption of ~ 17 g/L ethanol, and the final FAEEs titer reached a maximum of 9.97 g/L (including 1.3 g/L intracellular titers). The productivity was 90.6 mg/L/h, and the production yield was 86.1 mg/g.

To know whether there are any TAGs left in the cell mass, the lipid content was measured after batch and fed-batch cultivation. The data are shown in Additional file 1: Table S3. The result indicated that limited amounts of TAGs were detected in the intracellular lipids. Thus, we demonstrate that the Δ*ku70*-AbWS* is a robust FAEE producing platform with strong wax ester synthase activity.

## Discussion

Biodiesel can contribute significantly to the development of sustainable transportation fuels in the future [1], and biotechnology provides an alternative process, where the biodiesel can be produced from abundant biomass feedstocks using microorganisms. *R. toruloides*, a non-model oleaginous yeast, is an ideal organism to produce biodiesel because of its robust ability to accumulate single cell oils (SCOs) and capability to utilize various carbon sources [16]. Previously, this organism was used to produce biodiesel esters consisting of FAMEs through a “microbial oil process”, which involves the production of bio-oil by microbial cells and an additional transesterification reaction [24]. Moreover, a whole-cell catalyst technology was reported to convert 73% total lipids into FAEEs in the strain [20]. However, the catalytic mechanism was unclear and it was only hypothesized that the lipases on the membrane of lipid droplets might play an important role in the synthesis of FAEEs. All these reported processes had to lyse the cells for lipids extraction, which increased the production costs.

Recently, the emerging use of WSs highlighted the rational construction of microbial cell factories for directly producing FAEEs by fermentation in various hosts [5, 10–15, 25]. Inspired by these studies, we engineered a recombinant *R. toruloides* strain Δ*ku70*-AbWS* capable of directly producing FAEEs by fermentation. Compared with the previous studies [20, 24], which needed to separate the oil-accumulation and the esterification processes, the strategy reported here synchronized such two courses, thus avoiding additional esterification procedure. Moreover, it will be accessible to control the synthesis of FAEEs through metabolic engineering, which is not available in previous reports [20, 24].

Through fed-batch fermentation in a 1-L fermenter, we obtained a maximal FAEEs production of 9.97 g/L. This is to date the highest production titer in any yeast including the commonly used oleaginous yeast *Y. lipolytica* [15]. It is worth mentioning that the FAEEs produced by the engineered strain were mainly secreted outside the cell (8.6 g/L extracellular FAEEs were obtained). This is possibly due to its ability to assimilate hydrophobic carbon sources as substrates using an unknown transport system [16], which can work bidirectionally to secrete the product of FAEEs. For example, the transporter FATP1 can not only assimilate fatty acids, but also work as a fatty acid exporter [26]. The secretion may greatly reduce the production cost by avoiding product extraction as reported in *S. cerevisiae* [12, 13, 27]. Therefore, the engineered cell factory serves as a potential platform for the industrial production of FAEEs. In the future, genetic manipulation strategies such as overexpressing genes related to acetyl-CoA accumulation and eliminating competing pathways (peroxisome β-oxidation and TAG biosynthesis) are expected to further improve the production level.

While we have demonstrated that *R. toruloides* has great potential for large-scale production of biodiesels during this study, a major bottleneck to implement an economically feasible process is the additional cost associated with providing exogenous ethanol. Although it was reported that *R. toruloides* contained ethanol synthesis genes encoding PDC and ADH [28], it failed to produce ethanol under current experimental conditions (data not shown). Also there has been no report that *R. toruloides* has the ability to produce ethanol. To avoid the need to feed ethanol, two heterologous genes that encode PDC1 [29] and ADH4 [30] from *S. cerevisiae* were introduced to *R. toruloides* Δ*ku70*, with an aim to create an aerobic ethanol biosynthetic pathway. Unfortunately, the two corresponding enzymes could not be solubly expressed in the engineered strain based on Western blot (data not shown). In the future, more ethanol biosynthetic pathway genes from different ethanologenic organisms need to be evaluated and introduced to our engineered *R. toruloides* strain to construct an endogenously ethanol-producing pathway. Recently, this proposed strategy was demonstrated to be feasible in the oleaginous yeast *Y. lipolytica* for the synthesis of FAEEs without the addition of ethanol, but the titer of produced FAEEs was quite low (0.3 mg/L) due to the limited supply of ethanol [31]. Thus, further efforts to increase the production of ethanol is required. If successful, it will greatly promote the industrial development of biodiesel synthesis in the oleaginous yeasts.

## Conclusions

In this study, *R. toruloides* was engineered to produce FAEEs by fermentation for the first time by heterologously expressing the WS from *A. baylyi* ADP1. The optimal ethanol concentration for FAEEs production was proved to be 5%, which led to a maximum FAEEs titer of 0.82 g/L. To further improve the FAEEs production, the AbWS enzyme was modified by site-directed mutagenesis to change its substrate preferences, which significantly increased the production titer to 1.02 g/L. Finally, by carrying out fed-batch fermentation in a 1-L fermenter, the engineered strain was able to produce FAEEs up to a titer of 9.97 g/L. This is to date the highest FAEEs production level in a yeast host. Furthermore, the FAEEs produced by the strain were mainly secreted outside the cell, which can greatly save the costs for FAEE extraction. Overall, *R. toruloides* has the potential to become an excellent platform organism for industrial production of biodiesels and other fatty acid-derived green fuels or chemicals.

## Methods

### Strains and media

The parent strain in this study was *R. toruloides* Δ*ku70*, a derivative of *R. toruloides* IFO0880 with a deletion in the non-homologous end joining (NHEJ) gene, *ku70* [32]. *Agrobacterium tumefaciens* AGL-1 strain was used for transformation experiments [18, 33]. The *E. coli* DH5α was used for plasmid construction.

*R. toruloides* strains were routinely grown in liquid or solid yeast extract–peptone–dextrose (YPD) medium (10 g/L of yeast extract, 20 g/L of peptone, 20 g/L glucose) at 30 °C for strain construction and activation, and in fermentation medium (glucose 70.0 g/L, peptone 15.7 g/L, yeast extract 15.7 g/L) for both shake-flask batch fermentation and bioreactor fed-batch fermentation. *E. coli* strains were routine grown in Luria–Bertani broth (LB) medium at 37 °C. *A. tumefaciens* was grown at 28 °C in 2YT medium (16 g/L tryptone, 10 g/L yeast extract, 5 g/L NaCl). Kanamycin, ampicillin, hygromycin, cefotaxime and rifampicin were supplemented to the medium to a final concentration of 50 μg/mL, 100 μg/mL, 100 μg/mL, 300 μg/mL, and 25 μg/mL, respectively. Strain information used in this study are listed in Table 1.

### Plasmid construction and yeast transformation

Standard genetic manipulations were performed as previously described [35]. pKOCAR2 plasmid was used as the gene expression vector for yeast transformation [32]. The profile of the plasmid is shown in Additional file 1: Fig. S8. The plasmid contains a hygromycin selection cassette composed of a GPD1 promoter from *Rhodotorula graminis*, a codon optimized *hpt* gene and a Tsv40 terminator. A GPD1 promoter from *R. toruloides* and a T35s terminator was located downstream of the hygromycin selection cassette, where the exogenous gene can insert between them. The homologous sequences of Car2 gene (Car2 L and Car2 R) were located at both ends of the expression cassettes. The LB and RB sequences are the left and right border sequences of T-DNA, which can be identified by the virD complex in *A. tumefaciens* and transferred to *R. toruloides* through ATMT method.

The WS genes were codon optimized and synthesized by Nanjing GeneScript Biotech Co., Ltd (Nanjing, China). The optimized gene sequences and primers used in this study are listed in Additional file 1: Tables S2 and S4, respectively. To ligate these genes to pKOCAR2, the plasmid was firstly digested by EcoRV and NcoI, and then ligated with each of the DNA fragment by NEBuiler HiFi Assembly kit (New England Biolabs, USA), and finally yielded the recombinant plasmids p101, p102, p103, p104, and p105, respectively.

For enzyme engineering of AbWS, overlap-extension PCR was used for site-directed mutagenesis [36]. Two pairs of primer were designed to amplify the upstream and downstream DNA fragments, respectively. The modified nucleic base was contained in the primers. The mutated *AbWS* gene was ligated to the pKOCAR2 plasmid, yielding recombinant plasmid p1022. All plasmids used in this study are listed in Table 1.

The recombinant plasmids were transformed into *R. toruloides* by ATMT as described previously [37]. The pKOCAR2-derived plasmids were firstly electroporated into *A. tumefaciens* AGL-1. Then the *A. tumefaciens* strain harboring the corresponding plasmid was co-cultured with the *R. toruloides* Δ*ku70*, and the mixture was spread on YPD solid plate supplemented with hygromycin and cefotaxime for colony screening. Colonies appeared after 2 days.

### Fermentation conditions

Shake-flask batch fermentation was conducted in a 50-ml Erlenmeyer flask containing 10 ml of fermentation medium. The culture was inoculated with 10% seed culture, and performed in an orbital shaker at a rotary rate of 250 rpm at 30 °C. When the cell density reached around OD 2 (approximately 8–12 h after inoculation), exogenous ethanol was added to the culture to a final concentration of 1%, 3%, 5%, 7%, respectively. To facilitate the extraction process, 10% dodecane was added to the culture as reported previously [15]. Ethyl heptadecanoic was used as the internal standard.

High-density fermentation was conducted in a 1-L stirred bioreactor (Eppendorf, Germany) with an initial volume of 500 mL. The pH of the medium was monitored in real-time by a pH meter (Mettler-Toledo, Switzerland), and dissolved oxygen was monitored in real-time by an oxygen probe (Mettler-Toledo, Switzerland). The cultivation conditions were the same as what was previously described [21]. The inoculation volume was 50 mL, and the dissolved oxygen was set at 50% with an airflow rate of 1.25 vvm. The pH was maintained at 5.6 using 4 M NaOH. For batch fermentation, the initial glucose concentration was 70 g/L without feeding glucose during fermentation. For fed-batch fermentation, the initial glucose concentration was 70 g/L, and 50 mL glucose with a concentration of 500 g/L was fed to the culture when glucose concentration was below 10 g/L. 50 mL 50% ethanol was pumped to the fermenter at a flow rate of 1 mL/min to a final concentration of 5%. 50 mL dodecane together with ethyl heptadecanoate (C17:0) internal standard were injected to the fermenter through a syringe.

### Analytical methods

The growth curves of the *R. toruloides* strains were determined by measuring the cell density at 600 nm using a Genesys 20 spectrophotometer (Thermo Fischer Scientific Inc., USA). The dry cell weight (DCW) was determined by the previously described method [5]. The concentrations of glucose and ethanol were monitored by a LC-20A HPLC instrument (Shimadzu Inc., Japan) equipped with a RID detector as previously reported [15].

### Total lipids and FAEEs extraction and analysis

For extracellular FAEEs, the concentration was determined by directly injecting the dodecane layer to a SHIMADZU Japan GCMS-QP2010 PLUS mass spectrometer coupled with a SHIMADZU gas chromatograph (Shimadzu Inc., Japan). The ethyl heptadecanoic was added to the medium as the internal standard. The FAEEs dissolved in the dodecane layer were separated using a SHIMADZU 5MS capillary column (30 m × 0.25 mm I. D., 0.25 μm film thickness) as previously described [38].

For intracellular FAEEs, the concentration was measured by the following procedure. First, the total lipids were extracted. Cells were harvested and washed twice with double-distilled water (ddH_2_O), and then freeze-dried until the samples were dry. Lipids were extracted from the lyophilized cells as previously reported [39]. Ethyl heptadecanoic was added as an internal standard. Second, lipids were separated and fractionated. FAEE components in the total lipid extracts were separated by thin layer chromatography using TLC Silica gel 60 F254 plates as previously reported [12] (Merck, Darmstadt, Germany). The FAEE components were then scraped from the TLC plate and extracted by an organic solvent containing 3 mL hexane, 2 mL methanol and 2 mL ddH_2_O and vigorously vortexed for 1 h at room temperature. After centrifuging at 1676×*g* for 5 min, the organic phase was transferred to a new glass tube and dried under nitrogen. The residues were dissolved in 200 μL methanol: chloroform (95: 5) for GC–MS analysis.

To determine the fatty acid composition, total lipids were transmethylated and analyzed according to a previously reported method [40].

### WS assay

The samples were harvested from batch cultures during the mid-exponential phase (at OD 3) for enzyme activity measurement. The cultures were washed twice by 1 × phosphate buffer saline (PBS). Cell lysate was then prepared using a Fast Prep cell homogenizer (MP biomedicals, Solon, OH, USA). The proteins were quantified by a Bradford reagent (Sangon Biotech Co., Ltd. (Shanghai, China)). WS activity was determined by monitoring CoA release using Ellman’s reagent [5,5-dithio-bis (2-nitrobenzoic acid); DTNB] at 412 nm [38]. In vitro assays were performed in triplicate in 250 μL reaction system containing 125 mM sodium phosphate buffer (pH 7.4), 1.45% dimethyl sulfoxide (DMSO), 1 mM DNTB, 1 μM palmitoyl-CoA, 10 mM ethanol, and 10 ug of crude enzyme. Assay reactions were detected by a multimode plate reader (PerkinElmer Inc., USA).

### Western blot

The cells were sampled in the exponential growth period, and homogenized using a FastPrep instrument for 6 cycles at a speed of 6.0 m/s for 30 s each, with 5 min of interval. The suspensions were centrifuged at 15,000*g* for 30 min, and the supernatants were collected [34]. Then the total proteins were detected by sodium dodecyl sulfate–polyacrylamide gel electrophoresis (SDS-PAGE) with 12% polyacrylamide gels, which were then immediately transferred to a polyvinylidene difluoride (PVDF) membrane (BIO-RAD, USA). A Flag tag was added in the 5′ end of the *AbWS* gene. A monoclonal anti-FLAG M2 antibody was used for Western blot analysis as previously described [41], and the membrane was covered with electrochemiluminescence (ECL) plus solution for color reaction. Finally, the membrane was exposed to the Gel Imager System (Azure, USA) by the chemiluminescence mode.

## Supplementary Information

**Additional file 1.** Additional tables and figures.

## Data Availability

All data generated or analyzed during this study are included in this published article and its additional files.

## Abbreviations

GC–MS: Gas chromatography–mass spectrometry
TAG: Triglycerides
HPLC: High-performance liquid chromatography
LB: Luria–Bertani
ATMT: *A. tumefaciens-*Mediated transformation
DCW: Dry cell weight
TLC: Thin layer chromatography
PVDF: Polyvinylidene difluoride
