# Supplementary material for: Engineering oleaginous yeast Rhodotorula toruloides for overproduction of fatty acid ethyl esters
Source: Biotechnol Biofuels. 2021 May 8;14:115. doi: 10.1186/s13068-021-01965-3 (PMC8106135; doi:10.1186/s13068-021-01965-3)
Supplement: Supplementary file 1 — Additional file 1. Additional tables and figures. [file 13068_2021_1965_MOESM1_ESM.docx]

# Supplementary Information

**Engineering Oleaginous Yeast *Rhodotorula toruloides* for Overproduction of Fatty Acid Ethyl Esters**

Yang Zhang^1, †^, Jie Peng^1^^,^ **^†^**, Huimin Zhao^2,^ **^*^,** Shuobo Shi^1^^,^ **^*^**

^1^Beijing Advanced Innovation Center for Soft Matter Science and Engineering, College of Life Science and Technology, Beijing University of Chemical Technology, Beijing 100029, China

^2^Department of Chemical and Biomolecular Engineering, University of Illinois at Urbana-Champaign, Urbana, IL 61801, USA

^†^Yang Zhang and Jie Peng contributed equally to this work

**Correspondence:**

*E-mail: zhao5@illinois.edu.

*E-mail: shishuobo@mail.buct.edu.cn

Table S1. The information about the specific activities and FAEE production titers of the five WSs.

| Enzyme | Host | Enzyme activity (pmol/mg protein/min) | FAEE production titers (mg/L) | Reference |
| --- | --- | --- | --- | --- |
| WS from *A. baylyi* | *S. cerevisiae* | 4.6 ± 0.55 | 5.0 ± 0.8 | [1] |
| WS from *M. hydrocarbonoclasticus* | *S. cerevisiae* | 8.1 ± 1.87 | 6.3 ± 1.2 | [1] |
| WS from *R. opacus* | *S. cerevisiae* | 2.1 ± 0.37 | 2.1 ± 0.3 | [1] |
| WS from *M. musculus* | *S. cerevisiae* | 3.8 ± 0.51 | 1.3 ± 0.2 | [1] |
| WS from *P. arcticus* | *S. cerevisiae* | 5.9 ± 0.83 | 2.3 ± 0.4 | [1] |

Table S2. Primers used in present study.

| Primer names | Sequences | Purpose |
| --- | --- | --- |
| *AbWS*F | 5’−ATGGACTACAAGGACGACGACGACAAGCGTCCCCTCCACCCCAT−3’ | *AbWS* amplification |
| *AbWS*R | 5’−CTAGTTGGCGGTCTTGATGT−3’ | *AbWS* amplification |
| *MhWS*F | 5’−ATGGATTATAAAGATGACGATGACAAAAAACGTCTCGGCACCC−3’ | *MhWS* amplification |
| *MhWS*R | 5’−CTATTTACGGGCACGGGCAC−3’ | *MhWS* amplification |
| *MmWS*F | 5’−ATGGACTACAAGGACGACGACGACAAGTTCTGGCCCACCAAGAA−3’ | *MmWS* amplification |
| *MmWS*R | 5’−CTACTTGTCGTCGTCATCCT−3’ | *MmWS* amplification |
| *PaWSF* | 5’−ATGGACTACAAGGACGACGACGACAAGCGCCTCCTCACCGCCG−3’ | *PaWS* amplification |
| *PaWSR* | 5’−CTACTTGTCGTCGTCATCCT−3’ | *PaWS* amplification |
| *RoWSF* | 5’−ATGGACTACAAGGACGACGACGACAAGACCCAGACCGACTTCAT−3’ | *RoWS* amplification |
| *RoWSR* | 5’−CTACTTGTCGTCGTCATCCT−3’ | *RoWS* amplification |
| *AbWS-line1- F* | 5’−ACACCAGATCACTCACCATGATGGACTACAAGGACGACG−3’ | Point mutation of *AbWS* |
| *AbWS-line1- R* | 5’−CGCTTGGGCATCATACCCGAGATGATGTTGAG−3’ | Point mutation of *AbWS* |
| *AbWS-line2- F* | 5’−CTCAACATCATCTCGGGTATGATGCCCAAGCG−3’ | Point mutation of *AbWS* |
| *AbWS-line2- R* | 5’−CCCGGTCGGCATCTACGATATCTACTTGTCGTC−3’ | Point mutation of *AbWS* |
| *UCar2-F* | 5’-GACTGGACTACTGGCTCGTG-3’ | Verification of the insertion of WSs |
| *DCar2-R* | 5’-TGAAGAGCCTGTCCCATCTC-3’ | Verification of the insertion of WSs |

Table S3. The lipid content in the cell mass after batch or fed batch cultivation.

| Lipid contents (mg/g DCW) | Triglycerides | Diglycerides | Monoglycerides | Free fatty acids | FAEEs |
| --- | --- | --- | --- | --- | --- |
| Batch | 36.5 ± 5.2 | 7.4 ± 0.8 | - | 10.2 ± 1.3 | 25.4 ± 2.4 |
| Fed batch | 28.9 ± 3.8 | 4.5 ± 0.5 | - | 25.2 ± 4.0 | 37.7 ± 2.9 |

Table S4. Codon optimized sequences of wax ester synthase genes from different sources.

| Name | Optimized wax ester synthase gene from *A. baylyi* ADP1 (AbWS) |
| --- | --- |
| Sequence | atggactacaaggacgacgacgacaagcgtcccctccaccccatcgacttcatcttcctctcgctcgagaagcgccagcagcccatgcacgtcggtggcctcttcctcttccagatccccgacaacgcccccgacaccttcatccaggacctcgtcaacgacatccgtatctcgaagtcgatccccgtccctcccttcaacaacaagctcaacggcctcttctgggacgaggacgaggagttcgacctcgaccaccacttccgtcacatcgccctcccccaccccggtcgtatccgcgagctcctcatctacatctcgcaggagcactcgaccctcctcgaccgtgccaagcccctctggacctgcaacatcatcgagggtatcgagggcaaccgcttcgccatgtacttcaagatccaccacgctatggtcgacggtgtcgccggcatgcgtctcatcgagaagtcgctctcgcacgacgtcaccgagaagtcgatcgtccctccctggtgcgtcgagggcaagcgcgccaagcgtctccgtgagcccaagaccggcaagatcaagaagatcatgtcgggcatcaagtcgcagctccaggccacccccaccgtcatccaggagctctcgcagaccgtcttcaaggacatcggccgtaaccccgaccacgtctcgtcgttccaggccccctgctcgatcctcaaccagcgcgtctcgtcgtcgcgtcgcttcgccgcccagtcgttcgacctcgaccgtttccgcaacatcgccaagtcgctcaacgtcaccatcaacgacgtcgtcctcgccgtctgctcgggtgccctccgtgcctacctcatgtcgcacaactcgctcccctcgaagcccctcatcgctatggtccccgcctcgatccgtaacgacgactcggacgtctcgaaccgcatcaccatgatcctcgccaacctcgccacccacaaggacgaccccctccagcgtctcgagatcatccgtcgctcggtccagaactcgaagcagcgtttcaagcgcatgacctcggaccagatcctcaactactcggccgtcgtctacggtcccgccggcctcaacatcatctcgggtatgatgcccaagcgtcaggccttcaacctcgtcatctcgaacgtccccggtccccgtgagcccctctactggaacggtgccaagctcgacgccctctaccccgcctcgatcgtcctcgacggtcaggccctcaacatcaccatgacctcgtacctcgacaagctcgaggtcggcctcatcgcctgccgtaacgccctcccccgtatgcagaacctcctcacccacctcgaggaagagatccagctcttcgagggcgtcatcgccaagcaggaagacatcaagaccgccaacgattacaaggatgacgacgacaagtag |
| Name | Optimized wax ester synthase gene from *M. hydrocarbonoclasticus* DSM 8798 (MhWS) |
| Sequence | atgaaacgtctcggcaccctcgacgcctcgtggctcgccgtcgagtcggaggacacccccatgcacgtcggcaccctccagattttctcgctccccgagggtgcccccgagaccttcctccgcgacatggtcacccgtatgaaagaggccggtgacgtcgcccccccctggggttacaaactcgcctggtcgggtttcctcggtcgtgtcatcgcccccgcctggaaagtcgataaagacatcgacctcgactaccacgtccgtcactcggccctcccccgtcccggtggcgagcgtgagctcggtatcctcgtctcgcgcctccactcgaaccccctcgacttctcgcgtcccctctgggagtgccacgtcatcgagggtctcgaaaacaaccgcttcgccctctacaccaaaatgcaccactcgatgatcgacggtatctcgggcgtccgcctcatgcagcgtgtcctcaccaccgaccccgagcgttgcaacatgccccccccctggaccgtccgtccccaccagcgtcgcggtgccaaaaccgacaaagaggcctcggtccccgccgccgtctcgcaggctatggacgccctcaaactccaggccgacatggccccccgtctctggcaggccggtaaccgtctcgtccactcggtccgtcaccccgaggacggtctcaccgcccccttcaccggtcccgtctcggtcctcaaccaccgtgtcaccgcccagcgtcgtttcgccacccagcactaccagctcgaccgtctcaaaaacctcgcccacgcctcgggtggctcgctcaacgacatcgtcctctacctctgcggcaccgccctccgtcgcttcctcgccgagcagaacaacctccccgacacccccctcaccgccggcatccccgtcaacatccgtcccgccgacgacgagggcaccggcacccagatttcgttcatgatcgcctcgctcgccaccgacgaggccgaccccctcaaccgtctccagcagatcaaaacctcgacccgtcgcgccaaagagcacctccagaaactccccaaatcggccctcacccagtacaccatgctcctcatgtcgccctacatcctccagctcatgtcgggtctcggtggccgtatgcgtcccgtcttcaacgtcaccatctcgaacgtccccggtcccgagggcaccctctactacgagggtgcccgtctcgaagccatgtaccccgtctcgctcatcgcccacggtggtgccctcaacatcacctgcctctcgtacgccggttcgctcaacttcggtttcaccggctgccgtgacaccctcccctcgatgcagaaactcgccgtctacaccggcgaggccctcgacgagctcgaatcgctcatcctcccccccaaaaaacgtgcccgtgcccgtaaatag |
| Name | Optimized wax ester synthase gene from *Mus musculus* C57BL/ 6 (MmWS) |
| Sequence | atggactacaaggacgacgacgacaagttctggcccaccaagaaggacctcaagaccgctatggaggtcttcgccctcttccagtgggccctctcggccctcgtcatcgtcaccaccgtcatcatcgtcaacctctacctcgtcgtcttcacctcgtactggcccgtcaccgtcctcatgctcacctggctcgccttcgactggaagacccccgagcgtggtggccgtcgcttcacctgcgtccgtaagtggcgcctctggaagcactactcggactacttccccctcaagatggtcaagaccaaggacatctcgcccgaccgcaactacatcctcgtctgccacccccacggtctcatggcccactcgtgcttcggccacttcgccaccgacaccaccggtttctcgaagaccttccccggcatcaccccctacatgctcaccctcggtgccttcttctgggtccccttcctccgtgactacgtcatgtcgaccggttcgtgctcggtctcgcgttcgtcgatggacttcctcctcacccagaagggtaccggcaacatgctcgtcgtcgtcgtcggtggcctcgccgagtgccgttactcgacccccggttcgaccaccctcttcctcaagaagcgtcagggtttcgtccgtaccgccctcaagcacggcgtctcgctcatccccgcctacgccttcggtgaaaccgacctctacgaccagcacatcttcacccccggtggcttcgtcaaccgtttccagaagtggttccagaagatggtccacatctacccctgcgccttctacggtcgtggtctcaccaagaactcgtggggtctcctcccctactcgcagcccgtcaccaccgtcgtcggtgaacccctccccctccccaagatcgagaacccctcggaggagatcgtcgccaagtaccacaccctctacatcgacgccctccgcaagctcttcgaccagcacaagaccaagttcggtatctcggagacccaggagctcgtcatcgtcgattacaaggatgacgacgacaagtag |
| Name | Optimized wax ester synthase gene from *Psychrobacter arcticus* 273-4 (PaWS) |
| Sequence | atggactacaaggacgacgacgacaagcgcctcctcaccgccgtcgaccagctcttcctcctcctcgagtcgcgtaagcaccccatgcacgtcggtggcctcttcctcttcgagctccccgagaacgccgacatctcgttcgtccaccagctcgtcaagcagatgcaggactcggacgtccctcccaccttccccttcaaccaggtcctcgagcacatgatgttctggaaggaagacaagaacttcgacgtcgagcaccacctccaccacgtcgccctccccaagcccgcccgtgtccgcgagctcctcatgtacgtctcgcgcgagcacggtcgtctcctcgaccgtgccatgcccctctgggagtgccacgtcatcgagggtatccagcccgaaaccgagggctcgcccgagcgcttcgccctctacttcaagatccaccactcgctcgtcgacggtatcgccgccatgcgtctcgtcaagaagtcgctctcgcagtcgcccaacgagcccgtcaccctccccatctggtcgctcatggcccaccaccgcaaccagatcgacgccatcttccccaaggagcgctcggccctccgtatcctcaaggagcaagtctcgaccatcaagcccgtcttcaccgagctcctcaacaacttcaagaactacaacgacgactcgtacgtctcgaccttcgacgcccccaggtcgatcctcaaccgtcgcatctcggcctcgcgtcgcatcgccgcccagtcgtacgacatcaagcgcttcaacgacatcgccgagcgtatcaacatctcgaagaacgacgtcgtcctcgccgtctgctcgggtgccatccgtcgctacctcatctcgatggacgccctcccctcgaagcccctcatcgccttcgtccccatgtcgctccgtaccgacgactcgatcgccggtaaccagctctcgttcgtcctcgccaacctcggtacccacctcgacgaccccctctcgcgcatcaagctcatccaccgttcgatgaacaactcgaagcgtcgcttccgtcgcatgaaccaggcccaggtcatcaactactcgatcgtctcgtacgcctgggagggcatcaacctcgccaccgacctcttccccaagaagcaggccttcaacctcatcatctcgaacgtccccggttcggagaagcccctctactggaacggtgcccgtctcgagtcgctctaccccgcctcgatcgtcttcaacggtcaggccatgaacatcaccctcgcctcgtacctcgacaagatggagttcggtatcaccgcctgctcgaaggccctcccccacgtccaggacatgctcatgctcatcgaggaagagctccagctcctcgagtcggtttcgaaggagctcgagttcaacggtatcaccgtcaaggacaagtcggagaagaagctcaagaagctcgcccccgattacaaggatgacg acgacaagtag |
| Name | Optimized wax ester synthase gene from *Rhodococcus opacus* PD630 (RoWS) |
| Sequence | atggactacaaggacgacgacgacaagacccagaccgacttcatgtcgtggcgtatggaggaagaccccatcctccgttcgaccatcgtcgccgtcgccctcctcgaccgtcgccccgaccagtcgcgtttcgtcgacatgatgcgtcgcgccgtcgacctcgtccccctcttccgtcgcaccgccatcgaggaccccctcggtctcgcccctccccgttgggccgacgaccgtgacttcgacctctcgtggcacctccgtcgctacaccctcgccgagccccgtacctgggacggtgtcctcgacttcgcccgtaccgccgagatgaccgccttcgacaagcgtcgccccctctgggagttcaccatcctcgacggtctcaacgacggccgttcggccctcgtcatgaaggtccaccactcgctcaccgacggtgtctcgggcatgcagatcgcccgtgagatcgtcgacttcacccgcgagggtactccccgtcccggtcgtaccgaccgtgccaccgccgtcccccacggtggctcgtcgcgtcctccctcgcgtctctcgtggtaccgtgacaccgccgccgacgtcacccaccgtgccgccaacatcctcggtcgtaactcggtccgcctcgtccgtgcccccagggccacctggcgtgaggccaccgccctcgccggttcgaccctccgtctcacccgtcccgtcgtctcgaccctctcgcccgtcatgaccaagcgttcgacccgtcgccactgcgccgtcatcgacgtccccgtcgaggccctcgcccaagccgccgccgccgccgccggttcgatcaacgacgccttcctcgccgccgtcctcctcggtatggccaagtaccaccgtctccacggcgccgagatccgtgagctccgtatgaccctccccatctcgctccgtaccgagaccgaccccctcggtggcaaccgtatctcgctcgcccgtttcgccctccccaccgacatcgacgaccccgccgagctcatgcgtcgcgtccacgccaccgtcgacgcctggcgtcgcgagcccgccatccccttctcgcccatgatcgccggtgccgtcaacctcctccccgcctcgaccctcggcaacatgctcaagcacgtcgacttcgtcgcctcgaacgtcgccggttcgcccgtccccctcttcatcgccggttcggagatcctccactactacgccttctcgcccaccctcggttcggccttcaacgtcaccctcatgtcgtacaccacccagtgctgcgtcggcatcaacgccgacaccgacgccgtccccgacctcgccaccctcaccgagtcgctcgccgacggtttccgtgccgtcctcggtctctgcgccaagaccaccgacacccgtgtcgtcgtcgcctcggattacaaggatgacgacgacaagtag |

Figure S1. Comparison of FAEEs and total lipid compositions in Δ*ku70*-*AbWS* with additional 5% exogenous ethanol. In the total lipid composition column, C14 stands for methyl myristic; C16 stands for methyl palmitic, C18 stands for methyl stearic, C18:1 stands for methyl oleic and C18:2 stands for methyl linoleic. In the FAEE composition column, C14 stands for ethyl myristic; C16 stands for ethyl palmitic, C18 stands for ethyl stearic, C18:1 stands for ethyl oleic and C18:2 stands for ethyl linoleic.

Figure S2. TLC analysis of intracellular lipid samples in Δ*ku70*-*AbWS* under various ethanol concentrations. FAEE and TAG components were marked with arrows and emphasized with red boxes.

Figure S3. The extracellular FAEEs produced by the parent Δ*ku70* strain with an addition of exogenous ethanol with varying concentrations. C14, C16, C18, C18:1 and C18:2 stand for ethyl myristic, ethyl palmitic, ethyl stearic, ethyl oleic and ethyl linoleic, respectively.

Fig. S4 FAEEs titers of Δ*ku70*-*AbWS* in nitrogen limited medium under various ethanol concentrations. The left panel shows extracellular FAEEs titers and the right panel shows intracellular FAEEs titers. C14:0, C16:0, C18:0, C18:1 and C18:2 stand for ethyl myristic, ethyl palmitic, ethyl stearic, ethyl oleic and ethyl linoleic, respectively.

Fig. S5 The morphology and color of strain Δ*ku70,* Δ*ku70*-*AbWS*, and Δ*ku70*-*AbWS**.

Fig. S6 FAEEs titers of Δ*ku70*-*AbWS** in fermentation medium under various ethanol conditions. The left panel shows extracellular FAEEs titers and the right panel shows intracellular FAEEs titers. C14:0, C16:0, C18:0, C18:1 and C18:2 stand for ethyl myristic, ethyl palmitic, ethyl stearic, ethyl oleic and ethyl linoleic, respectively.

Fig. S7 FAEEs profiles in strain Δ*ku70*-*AbWS** and Δ*ku70*-*AbWS* when cultured in fermentation medium under 5% ethanol condition. C14:0, C16:0, C18:0, C18:1 and C18:2 stand for ethyl myristic, ethyl palmitic, ethyl stearic, ethyl oleic and ethyl linoleic, respectively.

Fig. S8 The profile of pKOCAR2 plasmid [2]. P_RgGPD_ stands for the GPD1 promoter from *Rhodotorula graminis*, and the P_GPD1_ stands for the GPD1 promoter from *R. toruloides*. *hpt* gene encodes the protein that resistant to hygromycin. The Car2 L and Car2 R are homologous sequences of Car2 gene.

**References**

[1] Shi S, Vallerodriguez J, Khoomrung S, Siewers V, Nielsen J. Functional expression and characterization of five wax ester synthases in *Saccharomyces cerevisiae* and their utility for biodiesel production. Biotechnol Biofuels*.* 2012; 5:7.

[2] Koh C, Liu Y, Moehninsi, Du M, Ji L. Molecular characterization of KU70 and KU80 homologues and exploitation of a KU70-deficient mutant for improving gene deletion frequency in *Rhodosporidium toruloides*. BMC Microbiol*.* 2014; 14:50.
